# Evaluation of MMP Inhibitors Isolated from *Ligustrum japonicum* Fructus

**DOI:** 10.3390/molecules24030604

**Published:** 2019-02-08

**Authors:** Hojun Kim, Fatih Karadeniz, Chang-Suk Kong, Youngwan Seo

**Affiliations:** 1 Division of Marine Bioscience, College of Ocean Science and Technology, Korea Maritime and Ocean University, Busan 49112, Korea; badguy47@naver.com; 2 Marine Biotechnology Center for Pharmaceuticals and Foods, Silla University, Baegyang-daero 700beon-gil 140, Sasang-gu, Busan 46958, Korea; karadenizf@outlook.com (F.K.), cskong@silla.ac.kr (C.-S.K.); 3 Department of Food and Nutrition, College of Medical and Life Sciences, Silla University, Baegyang-daero 700beon-gil 140, Sasang-gu, Busan 46958, Korea

**Keywords:** anti-invasive, HT-1080, *Ligustrum japonicum* fructus, MAPK, MMP

## Abstract

The current study investigated the ability of two secoiridoids, GL-3 (**1**) and oleonuezhenide (**2**), isolated from the fruits of *Ligustrum japonicum* to inhibit MMP-2 and -9 activity in phorbol 12-myristate 13-acetate (PMA)-induced HT-1080 human fibrosarcoma cells. Both compound**s** **1** and **2** were able to exert lowered gelatin digestion activity for MMP-2 and -9 tested by gelatin zymography via suppressing the release of MMPs to culture medium according to ELISA results. Treatment with compounds was also able to suppress the expression of both mRNA and protein levels of MMP-2 and -9. Action mechanism behind the MMP inhibitory effect of the compounds was suggested to be via MAPK pathway indicated by decreased levels of phosphorylated p38, ERK and JNK proteins evaluated employing immunoblotting. Compound **1** was shown to be slightly more active to inhibit MMP-2 and -9, however, compound **2** showed more regular dose-dependency during inhibition. In conclusion, this study suggested that GL-3 and oleonuezhenide were notable natural origin potent MMP inhibitors and could serve as lead compounds for development of anti-invasive MMP inhibitors against tumor metastasis.

## 1. Introduction

Matrix metalloproteinases (MMPs) are a family of Zn^2+^ dependent endopeptidases with more than 20 members which take roles in several diseases and complications such as chronic inflammation, periodontitis, chronic obstructive pulmonary disease, arteriosclerosis and arthritis [1,2,3]. MMPs are also known to be crucial in the progression, metastasis and invasion of the tumor cells owing to their ability to degrade and regenerate extracellular matrix [4,5]. This ability to degrade and reshape the extracellular matrix made MMPs also a key factor in the aging process of the skin and forming of the wrinkles which are mostly due to impaired collagen production and structure [6,7]. 

In particular, the secondary tumor growth where metastatic cancer cells from malignant tumors travel through the body via lymphatic system is closely-linked with the actions of several MMPs on the extracellular matrix of the target tissue for invasion [8,9]. It is mainly observed through MMP-mediated degradation of basement membrane proteins. The degrees of expression of two members of the MMP family, MMP-2 (gelatinase-A, 72 kDa) and MMP-9 (gelatinase-B, 92 kDa) were identified to be in closely related relationship with the metastasis and invasion ability of tumor cells, particularly secondary tumor growth [10,11]. As such, the primary cause of death in cancer patients is due to the secondary tumor growth rather than early diagnosed initial tumors. Hence, finding a substance that inhibits the enzymatic activity and/or production of MMPs is regarded as an important strategy towards overcoming cancer growth and linked mortality. For this reason, considerable efforts were directed into MMP inhibitory research and development [12,13,14]. Most of the reported MMP inhibitors are of synthetic origin and found through chemical synthesis pathways, however, research on MMP inhibitors from natural products has only been of increasing interest recently [15,16,17]. 

*Ligustrum japonicum* (Waxleaf privet) is an evergreen broad-leaved tree that is natively distributed in the western and southern coastal regions of Korea as well as the islands reachable from those shores. The small black oval fruit of this tree is a part of traditional folk medicine practices to cure liver and kidney problems and to treat hair whitening although it is also found to be toxic if consumed abundantly [18]. Studies revealed several bioactive properties of the *L japonicum* fruits such as antioxidant, anti-inflammatory, vascular relaxation, whitening and osteogenic stimulation effects [19,20,21]. In the process of developing a natural origin MMP inhibitor from terrestrial and marine plants, fruits of the *L. japonicum*, which were reported to possess MMP inhibitory properties in the extract level [19], were subjected to bioactivity-guided isolation to yield two bioactive secoiridoid glycosides. Current study investigated the effect of isolated compounds on the enzymatic activity and expression of MMP-2 and MMP-9 in HT-1080 human fibrosarcoma cell line known to have metastatic and invasive abilities and discussed the possibility of utilization towards the development of a natural MMP inhibitor against cancer metastasis. 

## 2. Results and Discussion

### 2.1. Structure Identification

**Figure 1:**
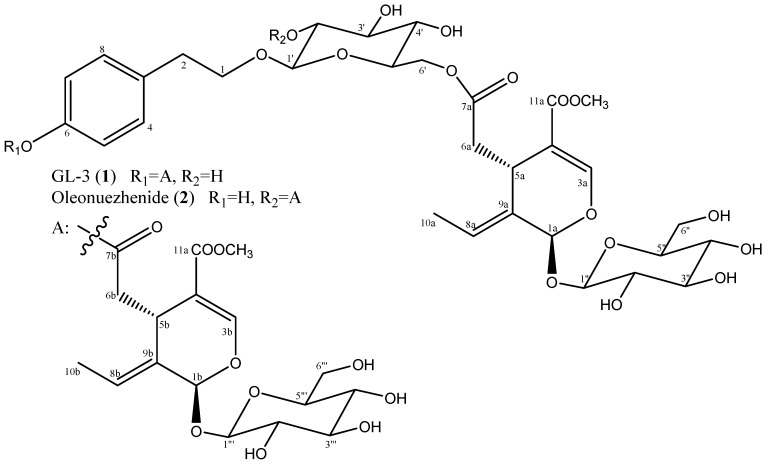
Chemical structures of isolated compounds GL-3 (**1**) and oleonuezhenide (**2**).

GL-3 (**1**) and oleonuezhenide (**2**) were previously isolated from *Fraxinus americana* and *Ligustrum japonicum,* respectively [22,23]. Chemical structures of these compounds were readily determined by a combination of spectroscopic analysis and comparison with data described in the literature (Figure 1). Their NMR spectral data (available in the Supplementary Material) were well matched with those reported in the same NMR solvent [24].

### 2.2. Inhibition of MMP-2 and MMP-9 Enzymatic Activity

**Figure 2:**
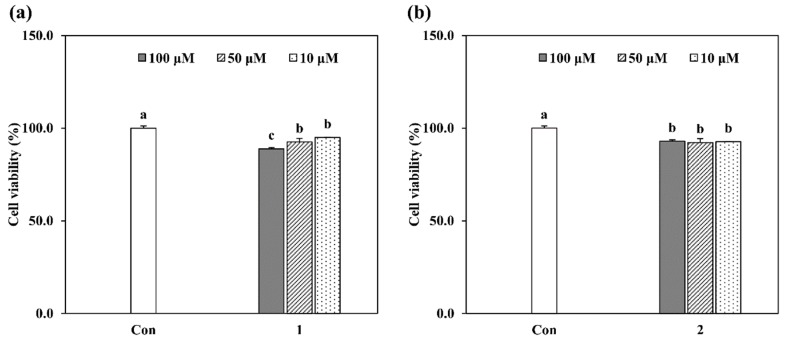
Effect of (**a**) GL-3 (**1**) and (**b**) oleonuezhenide (**2**) on the viability of HT-1080 human fibrosarcoma cells evaluated by MTT assay. Cell viability was given percentage of untreated control cells (Con). Values are mean ± SD of three independent experiments. ^a–c^ Means with different letters are significantly different at *p* < 0.05 level.

Prior to in vitro assays, biocompatibility of the isolated compounds GL-3 (**1**) and oleonuezhenide (**2**) was tested via evaluation of their toxic presence in HT-1080 human fibrosarcoma cell line. Cells treated with compounds **1** and **2** showed viability above 80% of untreated control cells at the concentrations 10, 50 and 100 µM (Figure 2). Further assays were conducted using these dose range and therefore, any MMP inhibitory effect of the compounds was defined not from cellular toxicity produced via compounds but other pathways stated.

**Figure 3:**
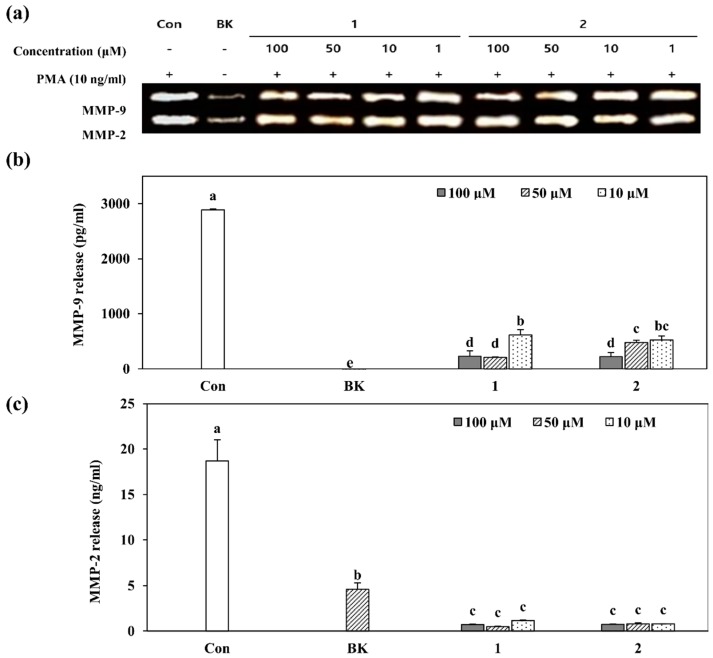
Gelatin zymography and ELISA showing the inhibitory effects of GL-3 (**1**) and oleonuezhenide (**2**) on enzymatic activity of MMP-2 and MMP-9 secreted by non-stimulated and PMA-stimulated HT-1080 human fibrosarcoma cells. (**a**) Cells were treated with indicated concentrations of compounds **1** and **2** for 24 h, and enzymatic activities of MMP-2 and MMP-9 from conditioned cell culture media were evaluated by electrophoresis of proteins on gelatin containing polyacrylamide gel. (**b**,**c**) Extracellular secretion levels of MMP-9 and MMP-2. Cells were treated with compounds **1** and **2** at indicated concentrations. Then, the cell culture medium was harvested and investigated by ELISA kits. Values are mean ± SD of three independent experiments. ^a–e^ Means with different letters are significantly different at *p* < 0.05 level. (BK: untreated unstimulated cells).

MMPs are involved in the metabolic pathways associated with cancer cell metastasis, oxidative stress, and fibrosis, among which MMP-2 and MMP-9 are known to be most closely related to tumor metastasis and invasion [10]. Enzymatic activity of the MMPs was tested using gelatin zymography assay [25]. As seen in Figure 3, the MMP-2 and MMP-9 activities were significantly increased in HT-1080 cells stimulated with PMA alone compared to that of untreated unstimulated cells. Treatment with both compounds **1** and **2** was able to lower the PMA-induced elevated activity of MMP-2 and MMP-9, however, the observed effect was not in a dose-dependent manner except the inhibition of MMP-9 release by compound **2** (Figure 3b).

To further confirm the inhibitory effects of compounds **1** and **2** on the release of MMPs to cell culture medium, conditioned cell culture media was analyzed by ELISA specific to MMP-2 and 9 (Figure 3b,c). Results were parallel to that of gelatin zymography as compounds **1** and **2** at 100 µM were able to lower the MMP-9 release to 228.6 pg/mL and 220.8 pg/mL, respectively, from 2888.6 pg/mL after PMA stimulation. Unlike gelatin degradation results, ELISA assay exhibited a significantly stronger decrease in MMP-2 release after treatment. Compound **1** and **2** treatment at 100 µM lowered the MMP-2 amount to 0.71 ng/mL and 0.75 ng/mL, respectively, from 17.1 ng/mL after PMA stimulation whereas untreated blank cells exhibited 4.6 ng/mL which was even higher than the treated groups for all treated concentrations. Inhibition of MMP-2 had higher rates compared to MMP-9 for both compounds. Inhibition of MMP-2 and MMP-9 release by compounds 1 and 2 was not dose-dependent where the efficiency was lower with higher doses in some groups indicating the doses were of above maximal inhibitory concentration. Both enzymatic activity and MMP release were inhibited in same manner with compounds **1** and **2**, non-dependent on treatment concentration, especially above a 50-µM concentration. This suggested a maximal inhibitory concentration (IC_50_) below 100 µM for compounds **1** and **2** which was comparable to doxycycline, a compound which is commonly used as positive control for experiments related to MMP inhibition. Doxycycline is a broad-spectrum antibiotic and important MMP inhibitor currently on the market as commercial drug. Reports indicated IC_50_ of doxycycline above 100 µM for inhibiting MMP-2 and MMP-9 enzymes [26]. Results indicated that the presence of compounds was able to lower the MMP-linked degradation of the gelatin, indicating direct inhibition of the secretion of the MMP-2 and -9 to the culture medium via interfering with intracellular MMP expression pathways according to ELISA results. 

### 2.3. Inhibition of MMP-2 and MMP-9 mRNA and Protein Expression

**Figure 4:**
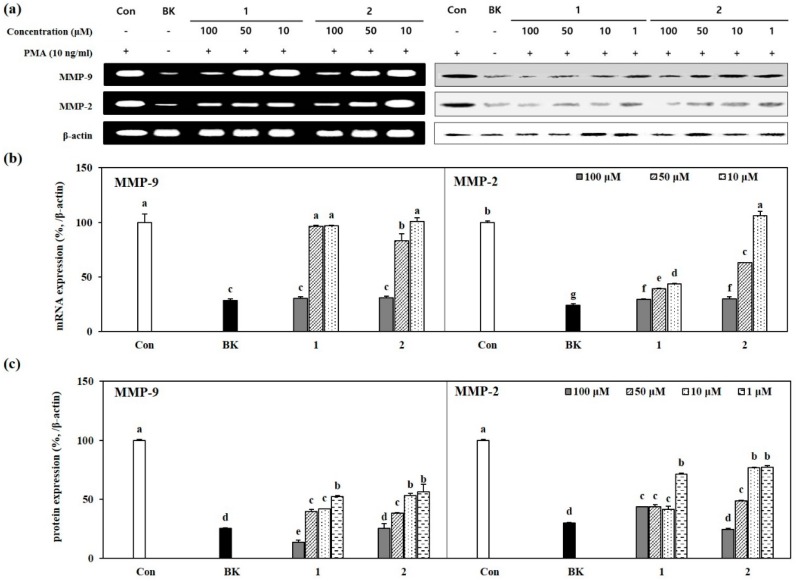
Effect of GL-3 (**1**) and oleonuezhenide (**2**) on the expression MMP-9 and MMP-2 mRNA and protein levels. (**a**) Cells were treated with indicated concentrations of compounds **1** and **2** prior to stimulation with PMA for 1 h and further incubated for 24 h. The expression levels of mRNA and proteins were detected on images of specific bands using RT-PCR and Western blotting, respectively. β-Actin was used as an internal standard. (**b**,**c**) Relative expression levels of MMP-9 and MMP-2 mRNA and proteins were given as percentage of stimulated untreated control cells (Con) after normalizing against internal control. Quantification of the bands was achieved by densiometric calculations. Values are mean ± SD of three independent experiments. ^a–g^ Means with different letters are significantly different at *p* < 0.05 level. (BK: untreated unstimulated cells).

In order to evaluate the inhibitory mechanism of compounds **1** and **2** on MMP-2 and MMP-9 suggested by gelatin zymography assay, both mRNA and protein expression levels were assessed by RT-PCR and Western blotting, respectively. Notable increase was observed for MMP-2 and -9 mRNA levels in PMA alone-stimulated cells compared to untreated unstimulated cells (Figure 4a,b). Treatment with 100 µM compounds **1** and **2** decreased the mRNA expression to prior levels before PMA stimulation, suggesting an inhibitory action on the expression of MMP-2 and -9 in the nucleus level. Similar results were observed for the protein levels (Figure 4a,c). Compound **1** treatment brought the MMP-9 protein levels even lower than that of untreated unstimulated cells. In terms of MMP-2 significant decrease in protein levels were also observed dose-dependently for compound **2**. Compound **1** treatment, however, showed same level of inhibition starting from 10 µM concentration up to 100 µM suggesting an inhibitory mechanism independent of the treatment dose. Overall, both compounds inhibited the protein and mRNA expressions of MMP-2 and -9, especially in terms of protein amounts. Dose-dependency was present, but MMP-9 mRNA expression was inhibited by compound **1** only at 100 µM treatment whereas compound **2** showed a slight inhibitory effect at 50 µM.

### 2.4. Inhibition of MAPK Pathway by Isolated Compounds

**Figure 5:**
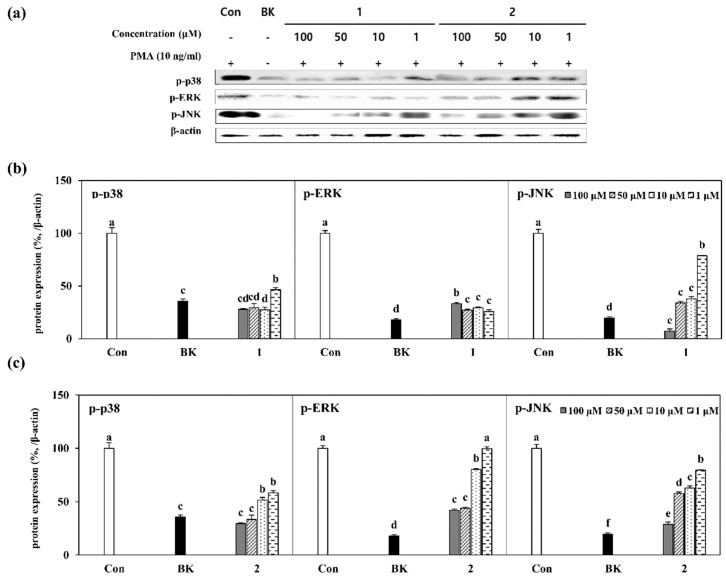
Effect of GL-3 (**1**) and oleonuezhenide (**2**) on the levels of phosphorylated (p-) p38, ERK and JNK proteins of MAPK pathway. (**a**) Cells were treated with indicated concentrations of compounds **1** and **2** prior to stimulation with PMA for 1 h and further incubated for 24 h. The levels of proteins were detected using Western blotting and given as images of protein-specific bands. β-Actin was used as an internal standard. (**b**,**c**) Relative expression levels of p-p38, p-ERK and p-JNK proteins were given as percentage of stimulated untreated control cells (Con) after normalizing against internal control. Quantification of the bands was achieved by densiometric calculations. Values are mean ± SD of three independent experiments. ^a–f^ Means with different letters are significantly different at *p* < 0.05 level. (BK: untreated unstimulated cells).

To understand the mechanism behind the inhibition of MMP-2 and -9 intracellular expression, a closely-linked signaling cascade, mitogen-activated protein kinase (MAPK), was investigated [27]. Phosphorylated levels of MAPK pathway regulators such as p38, ERK and JNK were determined as levels of protein amount by Western blotting. In regard to increased cellular activity following PMA stimulation MAPK pathway was expected to be activated along with the phosphorylation of p38, ERK and JNK. Several studies suggested a close relation between the inhibition of MAPK pathway activation and the downregulation of MMP-2 and -9 secretion as well as their expression [28]. Treatment with compounds **1** and **2** substantially lowered the protein expression levels of phosphorylated (p-) p38, p-ERK and p-JNK which were highly stimulated in PMA alone stimulated cells compared to untreated unstimulated group (Figure 5a). Quantification of the inhibition of MAPK pathway was also further confirmed by densiometric calculation of the immunoreactive bands for p-p38, p-ERK and p-JNK for compound **1** (Figure 5b) and **2** (Figure 5c). Results were in accordance with the mRNA expression results. Although compound **1** inhibited the phosphorylated p38, ERK and JNK levels, irregular inhibition patterns were observed for p38 and ERK (Figure 5b) except JNK where dose-dependent decrease was noted. On the other hand, compound **2** was able to inhibit all MAPK proteins tested dose-dependently, however, mostly in higher concentrations such as 50 and 100 µM.

Results clearly suggested an inhibitory effect against MMP-2 and -9 activity and expression for both compounds. Previous results reported strong bioactivities of derivatives of secoiridoids, especially secoiridoid glycosides [29,30,31]. 

## 3. Materials and Methods 

### 3.1. Apparatus and Reagents

The ^1^H- and ^13^C-NMR spectra were recorded on a Bruker Avance II NMR 900 spectrometer (Billerica, MA, USA). ^1^H and ^13^C spectra were measured using standard Bruker pulse sequence programs at 900 MHz and 225 MHz, respectively. All chemical shifts were recorded to the residual methanol-d_4_ (Cambridge Isotope Laboratories, Inc., Cambridge, MA, USA) peaks. 

### 3.2. Materials and Isolation

The fruits of *L. japonicum* were air-dried and extracted with dichloromethane (CH_2_Cl_2_) two times. The remaining residues were extracted with methanol (MeOH) twice in the equal amount as dichloromethane. The crude extracts (CH_2_Cl_2_ 9.12 g and MeOH 9.57 g) were concentrated under reduced pressure, combined and partitioned between CH_2_Cl_2_ and H_2_O. The organic dichloromethane layer was further partitioned between 85% aq. MeOH and *n*-hexane, and the aqueous layer was fractionated between *n*-BuOH and H_2_O, successively. The resulting four fractions were evaporated to dryness in vacuo, yielding the *n*-hexane (5.61 g), 85% aq. MeOH (4.63 g), *n*-BuOH (3.01 g), and H_2_O (5.15 g) fractions, respectively.

A portion of the *n*-BuOH (1.20 g) fraction was subjected to C_18_ silica gel column chromatography using stepwise gradient mixtures of H_2_O and MeOH (50%, 60%, 70%, 80%, 90% aq. MeOH, and 100% MeOH). The elution of *n*-BuOH fraction resulted in six sub-fractions, named rf 1 to 6, respectively. Among them, rf 2 was further subjected to HP20 resin column chromatography to obtain 6 fractions (100% H_2_O, 50% aq. MeOH, 50% aq. acetone, 100% MeOH, 100% acetone and washing), and named HP1-HP6. Among them, HP3 (50% aq. acetone) fraction was filtered by C_18_ silica gel with 20% aq. MeOH, and its filtrate was separated by reverse-phased HPLC (ODS-A, 47% aq. MeOH) to give two active components GL-3 (8.4 mg) and oleonuezhenide (9.0 mg).

#### 3.2.1. GL-3 (**1**)

^1^H-NMR (900 MHz, CD_3_OD) d 7.57 (1H, s, H-3a), 7.52 (1H, s, H-3b), 7.29 (2H, d, *J* = 9.0 Hz, H-4/-8), 6.98 (2H, d, *J* = 9.0 Hz, H-5/-7), 6.18 (1H, brq, *J* = 7.2 Hz, H-8b), 6.08 (1H, brq, *J* = 7.2 Hz, H-8a), 6.03 (1H, brs, H-1b), 5.92 (1H, brs, H-1a), 4.82 (1H, d, *J* = 7.2 Hz, H-1′’’), 4.80 (1H, d, *J* = 9.0 Hz, H-1”), 4.33 (1H, dd, *J* = 11.7, 2.0, H-6′), 4.20 (1H, dd, *J* = 11.7, 5.6 Hz, H-6′), 4.31(1H, d, *J* = 8.1 Hz, H-1′), 4.11 (1H, dd, *J* = 9.4, 4.4 Hz, H-5b), 4.02 (1H, dt, *J* = 12.0, 6.4 Hz, H-1), 4.00 (1H, dd, *J* = 9.1, 4.5, H-5a), 3.87 (1H, dd, *J* 11.7, 1.8 Hz, H-6”), 3.82(1H, d, *J* = 11.7 Hz, H-6′’’), 3.77 (1H, dt, *J* = 12.0, 8.1 Hz, H-1), 3.73 (3H, s, C-11a-OCH_3_), 3.68 (3H, s, C-11b-OCH_3_), 3.66 (1H, dd, *J* = 11.7, 6.3 Hz, H-6”), 3.63 (1H, dd, *J* = 11.7, 6.0, H-6′’’), 3.45 (1H, m, H-5′), 3.42-3.39 (2H, m, H-3′, -4′), 3.35-3.28 (8H, m, H-2′’, -2”’, -3′’, -3”’, -4′’, -4”’, -5′’, -5”’), 3.19 (1H, brt, *J* = 8.1 Hz, H-2′), 2.96 (1H, dd, *J* = 14.7, 5.0 Hz, H-6b), 2.93 (2H, t, *J* = 7.2 Hz, H-2), 2.75 (1H, dd, *J* = 14.4, 4.5 Hz, H-6a), 2.73 (1H, dd, *J* = 14.7, 8.1 Hz, H-6b), 2.50 (1H, dd, *J* = 14.4, 9.1 Hz, H-6a), 1.75 (3H, d, *J* = 7.2 Hz, H-10b), 1.72 (3H, d, *J* = 7.2 Hz, H-10a); ^13^C-NMR (225 MHz, CD_3_OD) δ 173.1 (C, C-7b), 171.7 (C, C-7a), 168.7 (C x 2, C-11a/-11b), 155.3 (CH, C-3b), 155.2 (CH, C-3a), 150.5 (C, C-6), 138.0 (C, C-3), 131.0 (CH x 2, C-4/-8), 130.6 (C, C-9b), 130.5 (C, C-9a), 125.2 (CH, C-8a), 125.0 (CH, C-8b), 122.5 (CH x 2, C-5/-7), 109.4 (C, C-4a), 109.3 (C, C-4b), 104.5 (CH, C-1′), 101.0 (CH, C-1′’), 100.9 (CH, C-1′’’), 95.3 (CH, C-1a or -1b), 95.2 (CH, C-1a or -1b), 78.41 (CH, C-5′’ or -5”’), 78.40 (CH, C-5′’ or -5”’), 77.94 (CH, C-3′’ or -3”’), 77.93 (CH, C-3′’ or -3′”), 77.91 (CH, C-3′), 75.2 (CH x 2, C-2′/-5′), 75.0 (CH, C-2′’ or -2”’), 74.8 (CH, C-2′’ or -2”’), 71.61 (CH_2_, C-1), 71.57 (CH, C-4′’ or -4”’), 71.51 (CH, C-4′’ or -4”’), 71.4 (CH, C-4′), 65.3 (CH_2_, C-6′), 62.72 (CH_2_, C-6′’ or -6”’), 62.66 (CH_2_, C-6′’ or 6”’), 52.0 (CH_3_ x 2, C-11a-OCH_3_/-11b-O CH_3_), 41.2 (CH_2_, C-6b), 41.1 (CH_2_, C-6a), 36.6 (CH_2_, C-2), 31.80 (CH, C-5a or -5b), 31.78 (CH, C-5a or -5b), 13.82 (CH_3_, C-10a or 10b), 13.75 (CH_3_, C-10a or 10b); FABMS *m/z* 1095 [M + Na]^+^.

#### 3.2.2. Oleonuezhenide (**2**)

^1^H-NMR (900 MHz, CD_3_OD) 7.52 (2H, s, H-1a/1b), 7.00 (2H, d, *J* = 9.0 Hz, H-2/-6), 6.67 (2H, *J* = 9.0 Hz, H-3/-5), 6.10 (2H, q, *J* = 8.1 Hz, H-8a/-8b), 5.95 (1H, brs, H-1b), 5.92 (1H, brs, H-1a), 4.80 (2H, d, *J* = 8.1 Hz, H-1”/-1”’), 4.65 (1H, dd, *J* = 9.0, 8.1 Hz, H-2′), 4.43 (1H, d, *J* = 8.1 Hz, H-1′), 4.34 (1H, dd, *J* = 11.7.0, 2.0 Hz, H-6′), 4.21 (1H, dd, *J* = 11.7, 5.8 Hz, H-6′), 4.01 (1H, dd, *J* = 9.5, 5.2 Hz, H-5a), 4.00 (1H, dd, *J* = 9.5, 4.4 Hz, H-5b), 3.94 (1H, dt, *J* = 9.4, 8.0 Hz, H-1), 3.893 (1H, dd, *J* = 11.4, 2.0 Hz H-6” or -6′”), 3.890 (1H, dd, *J* = 11.4, 2.0 Hz H-6” or -6′”), 3.72 (3H, s, H-11a-OCH_3_), 3.69 (3H, s, H-11b-OCH_3_), 3.66 (2H, m, H-6”/-6′”), 3.60 (1H, dt, *J* = 9.4, 8.0 Hz, H-1), 3.51 (1H, t, *J* = 8.6 Hz, H-3′), 3.48 (1H, ddd, *J* = 9.2, 5.9, 2.8 Hz, H-5′), 3.26-3.42 (9H, m, H-4′, -2”, -3”, 4”, 5”, 2′”, 3′”, 4′”, 5′”), 2.75 (1H, dd, *J* = 14.5, 5.2, H-6a), 2.74 (2H, t, *J* = 8.0 Hz, H-2), 2.64 (1H, dd, *J* = 14.5, 4.4 Hz, H-6b), 2.51 (1H, dd, *J* = 14.5, 9.5 Hz, H-6b), 2.50 (1H, dd, *J* = 14.5, 9.5 Hz, H-6a), 1.75 (3H, dd, *J* = 7.2, 1.8 Hz, H-10b), 1.73 (3H, dd, *J* = 7.2, 1.8 Hz, H-10a); ^13^C-NMR (300 MHz, CD_3_OD) δ 173.0 (C, C-7a), 172.1 (C, C-7b), 168.8 (C, C-11a), 168.7 (C, C-11b), 156.8 (C, C-6), 155.3 (C, C-3a), 155.2 (C, C-3b), 131.0 (CH x 2, C-4/-8), 130.9 (C, C-3), 130.5 (C, C-9a), 130.3 (C, C-9b), 125.1 (CH, C-8b), 124.9 (CH, C-8a), 116.1 (CH x 2, C-5/-7), 109.4 (C x 2, C-4a/-4b), 102.1 (CH, C-1′), 101.0 (CH, C-1”), 100.9 (CH, C-1′”), 95.5 (CH, C-1b), 95.2 (CH, C-1a), 78.44 (CH, C-3” or 3′”), 78.42 (CH, C-3” or 3′”), 77.96 (CH, C-5” or -5′”), 77.94 (CH, C-5” or -5′”), 75.7 (CH, C-3′), 75.28 (CH, C-2′ or -5′), 75.24 (CH, C-2′ or -5′), 74.76 (CH, C-2” or -2′”), 74.74 (CH, C-2” or -2′”), 71.73 (CH_2_, C-1), 71.69 (CH, C-4”), 71.55 (CH, C-4′”), 71.50 (CH, C-4′), 64.8 (CH_2_, C-6′), 62.7 (CH_2_ x 2, C-6”/-6′”), 52.0 (CH_3_ x 2, C-11a-OCH_3_/-11b-OCH_3_), 41.2 (CH_2_, C-6a), 40.9 (CH_2_, C-6b), 36.2 (CH_2_, C-2), 31.8 (CH, C-5a), 31.5 (CH, C-5b), 13.9 (CH_3_, C-10b), 13.8 (CH_3_, C-10a); FABMS *m/z* 1095 [M+Na]^+^.

### 3.3. Cell Culture and Cytotoxicity Determination 

In vitro bioactivity assays were carried out using HT-1080 human fibrosarcoma cell line. Cells were cultured with Dulbecco’s modified eagle medium (DMEM, Gibco-BRL, Gaithersburg, MD, USA) including 10% fetal bovine serum (FBS), 2 mM glutamine and 100 μg/mL penicillin-streptomycin (Gibco-BRL, Gaithersburg, MD, USA) in cell culture flasks (T-75, Nunc, Roskilde, Denmark) and kept at humidified incubators at 37 °C with 95% air/5% CO_2_ atmosphere. Unless specified otherwise, cell culture medium was changed with fresh one every 72 h.

Possible cytotoxic presence of the samples was assessed by a colorimetric cell viability assay where cells were grown in 96-well plates following seeding at a 5 × 10^3^ cells/well density. Samples with different concentrations (5, 10, 20, 50 and 100 μg/mL) were introduced to the wells following 24 h incubation after the cell culture medium was aspired from wells and cells were washed with fresh medium prior to treatment. Sample treated wells were incubated for 24 or 48 h and 100 μL of MTT solution (1 mg/mL) was added to the wells following the cell culture removal and washing with fresh medium. One hundred microliters of DMSO was introduced to each well following 4 h incubation with the purpose of solubilizing the formazan crystals. Quantity of the formazan crystals for each well was determined by the reading of absorbance values at 540 nm using a GENios^®^ microplate reader (Tecan Austria GmbH, Grödig, Austria). Change of viability of the HT-1080 cells in regard to sample treatment was defined proportional to absorbance value considering the ability of the viable cells to convert MTT into formazan crystal. Determination of the cytotoxicity of the samples was performed via comparing untreated control against sample treated cells and dose-dependent changes in cell viability were demonstrated as percentage of control cells.

### 3.4. Determination of MMP Activity by Gelatin Zymography

Effect of samples on the enzymatic activity of MMP-2 and MMP-9 were evaluated by gelatin zymography. Slightly modified method of Bae et al. [25] for the gelatin zymography was adopted. Prior to sample treatment, cells were stimulated with phorbol 12-myristate 13-acetate (PMA, 10 ng/mL) in order to enhance MMP expression and secretion into conditioned cell culture media which later was harvested and used for the determination of the MMP activity on the gels. Cell culture medium-loaded gels were subjected to substrate-gel electrophoresis and digestion of the gelatin by MMPs was further stimulated by maintaining gels in a buffer solution (10 mM CaCl_2_, 50 mM Tris–HCl and 150 mM NaCl) at 37°C for 48 h. Enzymatic activities were detected and graded as clear zones digested by MMP-2 and MMP-9 activity visible after Coomassie Blue staining of the gels and observed with CAS-400SM Davinch-Chemi imager^TM^ (Davinch-K, Seoul, Korea).

### 3.5. Enzyme-Linked Immunosorbent Assay

HT-1080 cells were pre-incubated in 6-well plates for 24 h and the cells were washed with PBS followed by PMA stimulation. After PMA stimulation, the cells were incubated in the presence or absence of bioactive compounds isolated from *L. japonicum* fructus for 24 h. MMP-2 and MMP-9 proteins in culture supernatants were analyzed by ELISA kit (R&D systems, Inc., Minneapolis, MN, USA) per manufacturer’s instructions.

### 3.6. Reverse Transcription-Polymerase Chain Reaction Analysis

Expression of MMP-2 and MMP-9 mRNA was assessed by reverse transcription-polymerase chain reaction (RT-PCR) analysis. Established protocols were performed with previously reported forward and reverse primers [25]. Total RNA was isolated from the sample treated or untreated cells with the help of Trizol reagent and 2 μg of isolated total RNA was used for cDNA synthesis and later amplification of the target genes via reverse transcription system (Promega, Madison, WI, USA) employing manufacturer’s instructions. Expression of target mRNAs was assessed by examining the bands of the amplified products on the EtBr-stained gels under UV light using a CAS-400SM Davinch-Chemi imager^TM^ (Davinch-K, Seoul, Korea). Quantification of the bands was carried out via densiometric calculation using AlphaEase^®^ gel image analysis software (Alpha Innotech, San Leandro, CA, USA).

### 3.7. Western Blot Analysis

Protein expression levels of MMP-2 and MMP-9 in treated and untreated HT-1080 cells were detected by immunoblotting using standard procedures previously reported [25]. Concisely, treated and untreated HT-1080 cells were transferred into tubes containing RIPA lysis buffer (Sigma-Aldrich Corp., St. Louis, MO, USA) and vigorously shaken for 30 min at 4 °C. A portion (35 μg) of the obtained lysates was loaded to 12% SDS-polyacrylamide gel. Gels were subjected to electrophoresis for separation of the cell lysates. Separated proteins were then electrotransferred onto a polyvinylidene fluoride membrane (Amersham Pharmacia Biosciences., England, UK) from gels and the target proteins were detected with an electrochemiluminescence kit (Amersham Pharmacia Biosciences, England, UK) after the incubation of the membranes with specific antibodies for target proteins and secondary antibodies for detection. Immunoreactive protein bands were visualized using CAS-400SM Davinch-Chemi imager^TM^ (Davinch-K, Seoul, Korea) and densiometric quantification of the bands were performed with AlphaEase^®^ gel image analysis software (Alpha Innotech, San Leandro, CA, USA).

### 3.8. Statistical Analysis

Quantified values were given as a mean of the results of experiments done in triplicate. Relative differences of data were determined by one-way ANOVA followed up by Duncan’s multiple range post hoc test using SAS v9.1 (SAS Institute Inc., Cary, NC, USA) software. Any statistically meaningful difference was defined at *p* < 0.05 level.

## 4. Conclusions

Current study was the first report of their MMP inhibitory effect on MMP-2 and -9 to the best of our knowledge. Both compounds exhibited strong inhibitory effects towards the enzymatic activity of MMP-2 and -9 along suppressed expression for mRNA and protein levels. Examination of the phosphorylated MAPK protein levels exhibited that both compounds might regulate the MMP expression via downregulation of MAPK pathway. In addition, chemical structures of the secoiridoid glycosides were previously reported to possess enzyme inhibitory properties to various types of MMPs [32,33,34]. In this context, both compounds **1** and **2** were suggested to be potential MMP inhibitors with strong suppressing effect on the expression of MMP-2 and -9 via MAPK pathway. Irregular inhibitory patterns in terms of dose-dependency might suggest utilization of the compounds as pre-cursors for further development of stable and clinically active MMP inhibitors against cancer metastasis and invasion.

## Supplementary Materials

The following are available online, Figure S1: ^1^H-NMR Spectrum of GL-3 (**1**) (MeOH-d4, 900 MHz), Figure S2: ^13^C-NMR Spectrum of GL-3 (**1**) (MeOH-d_4_, 900 MHz), Figure S3: ^1^H-NMR Spectrum of oleonuezhenide (**2**) (MeOH-d_4_, 900 MHz), Figure S4: ^13^C-NMR Spectrum of oleonuezhenide (**2**) (MeOH-d_4_, 900 MHz).
