# Supplementary material for: Evaluation of MMP Inhibitors Isolated from Ligustrum japonicum Fructus
Source: Molecules. 2019 Feb 8;24(3):604. doi: 10.3390/molecules24030604 (PMC6384611; doi:10.3390/molecules24030604)
Supplement: Supplementary file 1 [file molecules-24-00604-s001.pptx]

## Slide 1
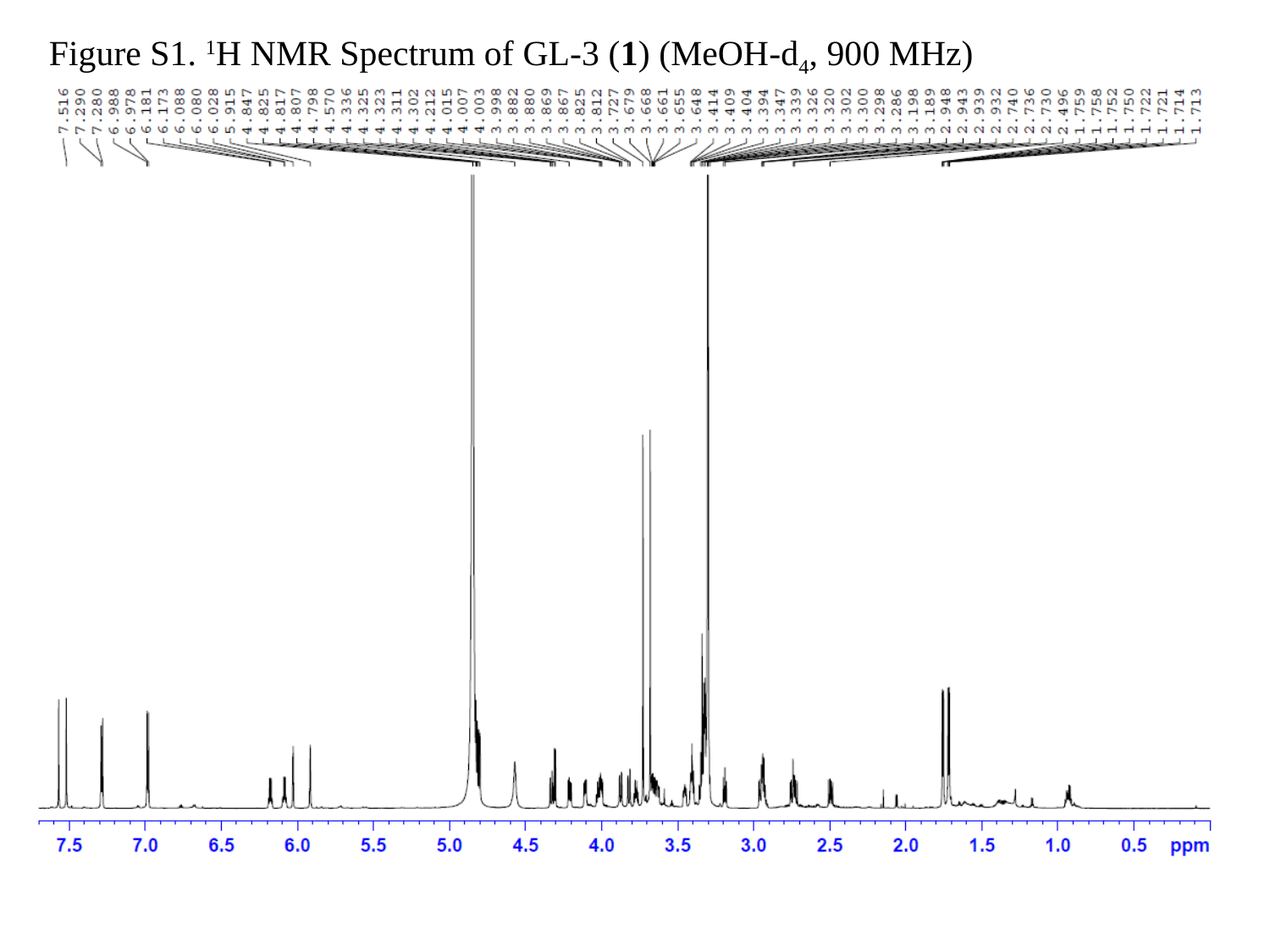

Figure S1. 1H NMR Spectrum of GL-3 (1) (MeOH-d4, 900 MHz)

## Slide 2
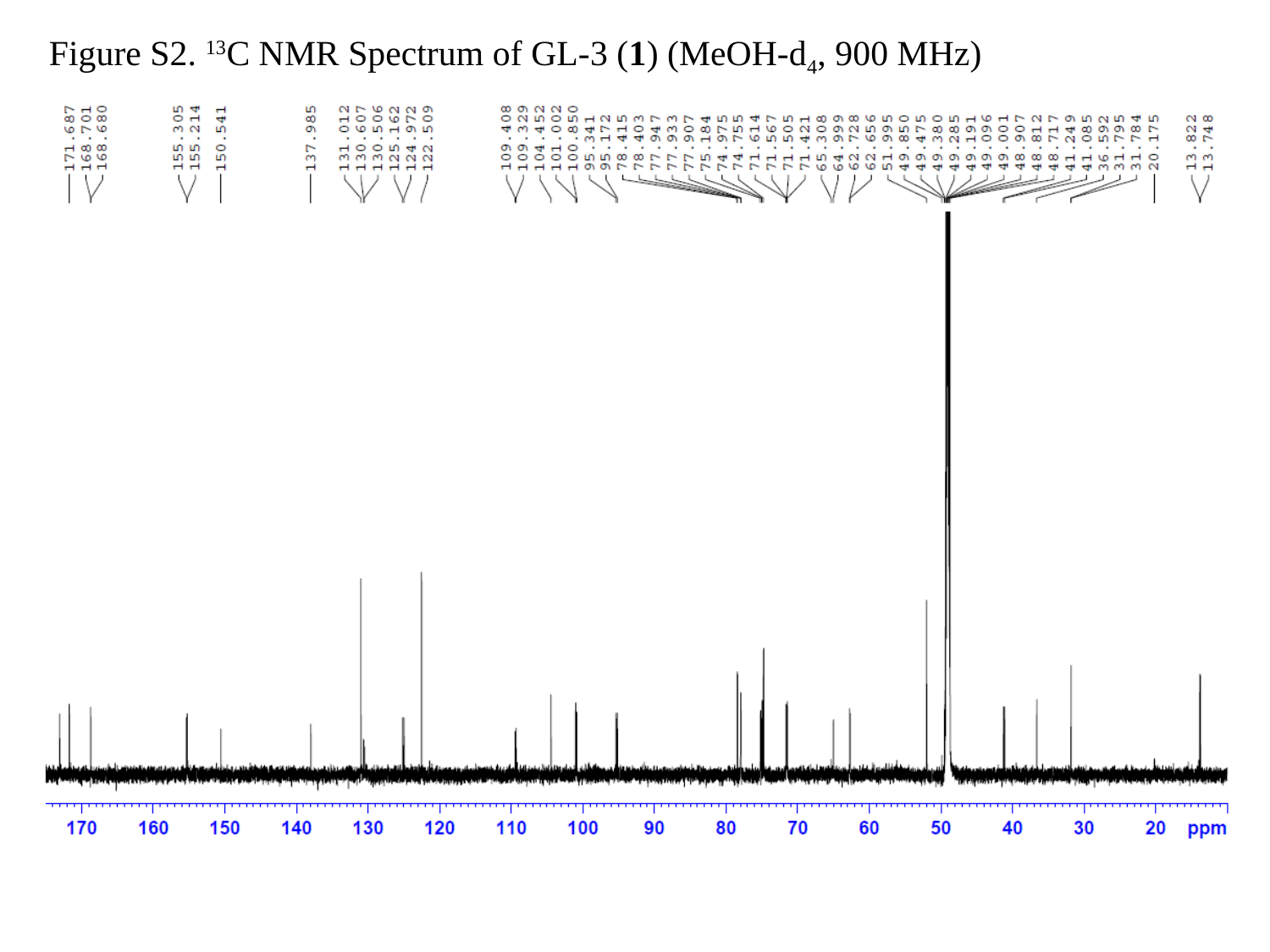

Figure S2. 13C NMR Spectrum of GL-3 (1) (MeOH-d4, 900 MHz)

## Slide 3
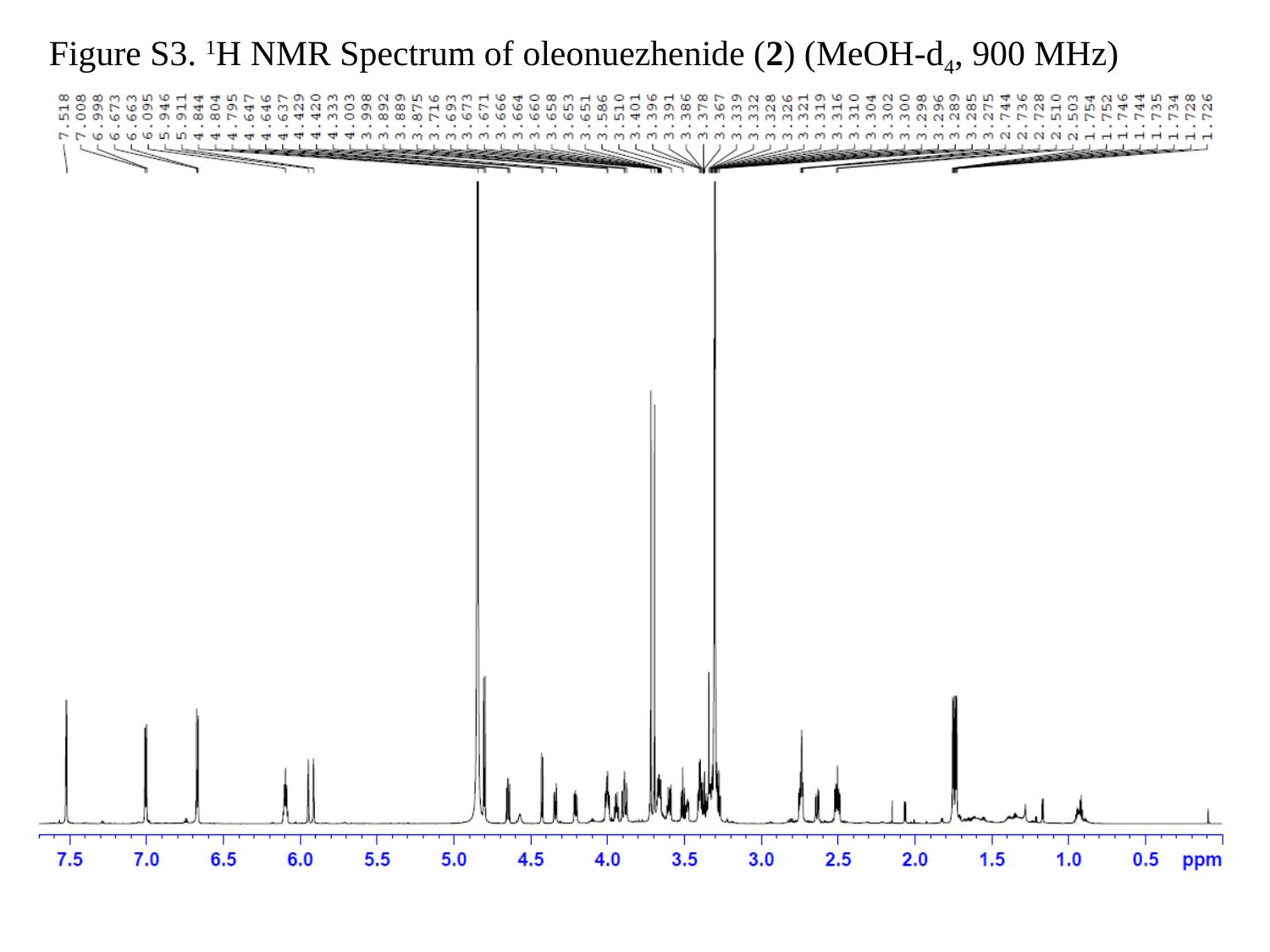

Figure S3. 1H NMR Spectrum of oleonuezhenide (2) (MeOH-d4, 900 MHz)

## Slide 4
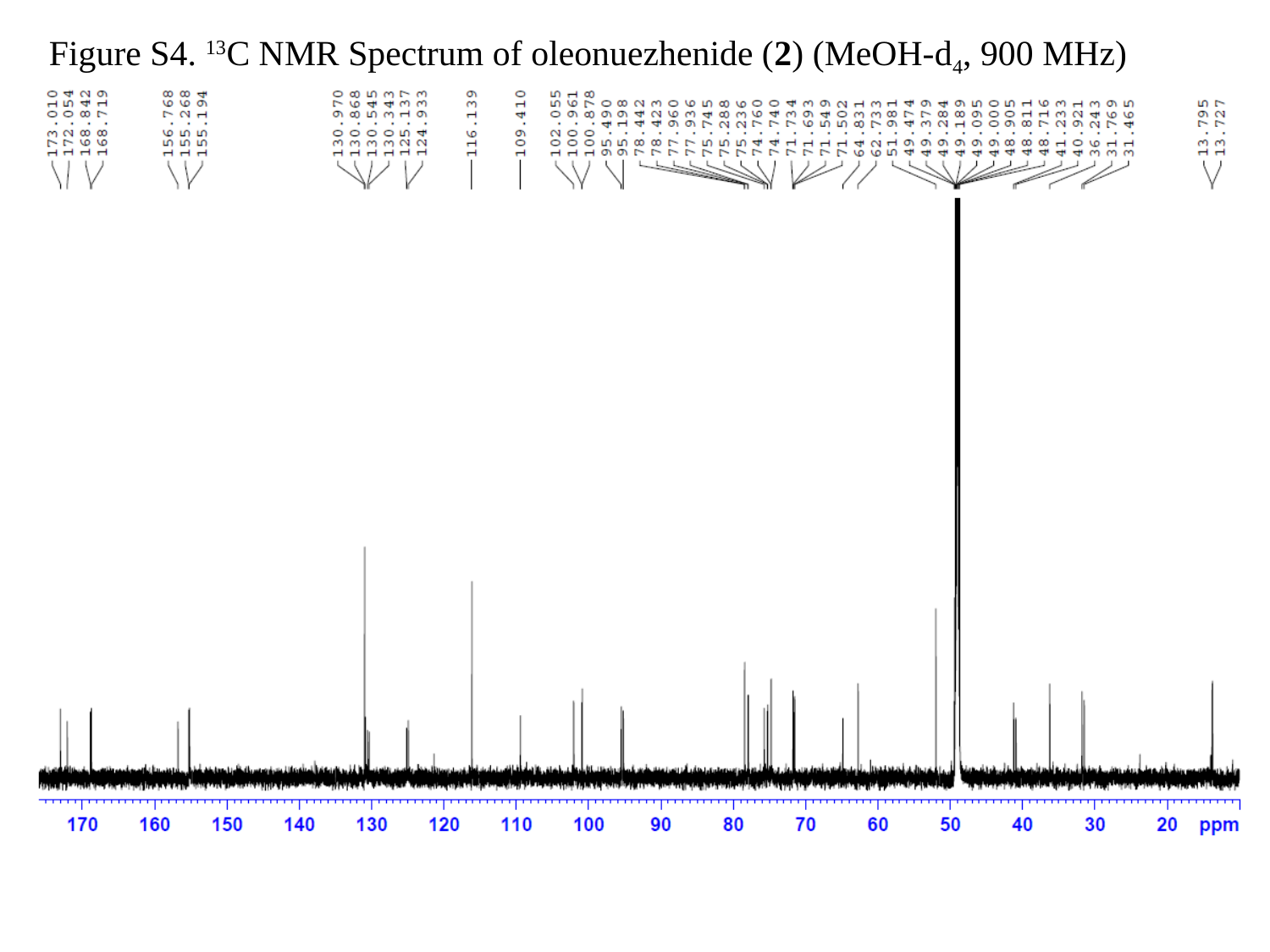

Figure S4. 13C NMR Spectrum of oleonuezhenide (2) (MeOH-d4, 900 MHz)
